# Real-Time Monitoring the Growth of Epitaxial Co_*x*_Fe_3−*x*_O_4_ Ultrathin Films on Nb-Doped SrTiO_3_(001) via Reactive Molecular Beam Epitaxy by Means of Operando HAXPES

**DOI:** 10.3390/ma15072377

**Published:** 2022-03-23

**Authors:** Kevin Ruwisch, Tobias Pohlmann, Florian Bertram, Christoph Schlüter, Andrei Gloskovskii, Karsten Küpper, Joachim Wollschläger

**Affiliations:** 1 Department of Physics, Osnabrück University, 49076 Osnabrück, Germany; tobias.pohlmann@uos.de (T.P.); kkuepper@uos.de (K.K.); jwollsch@uos.de (J.W.); 2 Deutsches Elektronen-Synchrotron (DESY), Photon Science, 22607 Hamburg, Germany; florian.bertram@desy.de (F.B.); christoph.schlueter@desy.de (C.S.); andrei.hloskovsky@desy.de (A.G.)

**Keywords:** cobalt ferrite, ultrathin films, operando HAXPES, growth study

## Abstract

In this work, we present a comprehensive study on real-time monitoring the growth of epitaxial Co_*x*_Fe_3−*x*_O_4_ thin films grown on SrTiO_3_(001) substrates via reactive molecular beam epitaxy. The growth process was monitored during evaporation by means of time resolved operando hard X-ray photoelectron spectroscopy (HAXPES). We prepared ultrathin ferrite films using different oxygen partial pressures, showing pure metallic, light oxidic, and cobalt ferrite-like growth. Additional X-ray diffraction measurements confirm HAXPES results.

## 1. Introduction

Transition metal oxides exhibit numerous properties, such as competition of spin, charge, and orbital degrees of freedom. More specifically, a lot of ferrites show a high Curie temperature $T_{C}$ and a high magnetic saturation moment. Hence, these ferrites might be excellent materials for applications in the fields of spintronics [1,2,3] or spincaloritronics [4]. Many of these Fe based oxides exhibit varying electronic and magnetic properties and crystallize in the inverse spinel structure. Examples include the semimetallic and ferrimagnetic Fe_3_O_4_, the semiconducting γ−Fe_2_O_3_, and the more insulating ferrimagnets NiFe_2_O_4_ and CoFe_2_O_4_ (CFO). The latter mentioned oxides, i.e., NiFe_2_O_4_ and CoFe_2_O_4_ are in particular interesting for spin-filter applications [5,6,7,8]. The aim for spin-filter applications is generating highly spin-polarized currents due to spin polarization dependent tunneling of electrons. Because the structural quality of the tunnel barrier and its interfaces significantly affects the spin filter efficiency and is important for the transmission of spin currents, ferrites must be fabricated as thin films with low defect density, which appears to be one of the major obstacles as spintronic devices with NiFe_2_O_4_ and CoFe_2_O_4_ electrodes did not prove to be very successful yet. Only for CoFe_2_O_4_ a room temperature spin filtering effect with a spin-polarization value of −8% was obtained up to now [7]. These shortcomings have been associated to the structural quality and the chemical properties of the ferrite ultra thin films right at the substrate interface. Antiphase boundaries (APBs) and other interface effects might lead to the formation of a magnetic dead layer, for instance [7,9]. Besides the above mentioned effects Ti diffusion into CoFe_2_O_4_ thin films grown on SrTiO_3_(001) substrates has been reported [10]. In addition to the interfacial effects mentioned above, the magnetic and structural properties strongly depend on other parameters such as thin film thickness [11], or growth conditions such as growth temperature or oxygen pressure [12]. Since ferrites are known to form different morphologies, such as rock salt and spinel structure, it is of utmost interest to study the electronic structure during growth. Hence, we obtain information on the different stages of growth in real time and therefore develop a deeper understanding of the initial stages of the thin film formation. Moreover, for spin-filters, it is crucial to fabricate stochiometric CoFe_2_O_4_. Metallic Fe and Co is not used in the application, so here we are looking for the right parameters to produce stoichiometric CoFe_2_O_4_ and observe the evolution of the film during fabrication.

In the present work we perform a real time monitoring of the epitaxial growth of thin Co_*x*_Fe_3−*x*_O_4_ films on Nb-doped SrTiO_3_(001) substrates with varying partial oxygen pressures employing hard X-ray photoelectron spectroscopy (HAXPES). Peak positions and satellites in the spectra of photoelectron spectroscopy measurements are unique features for the oxidation state. We exploit this feature to make deductions about the chemical composition. HAXPES is a powerful tool to investigate the electronic and chemical properties not only on the surface near regions of a sample in question but also in deeper layers due to excitation energies of several keV. Hence, we are able to collect valuable information from the whole ultra thin film during the overall growth process. We demonstrate that the different partial oxygen pressures lead to the formation of different Fe and Co valence states and final compounds but also that the Fe and Co oxidation processes may continue longer than the thin film deposition process itself.

## 2. Materials and Methods

Film preparation and analysis were carried out at beamline P22 of synchrotron radiation source PETRA III at the Deutsches Elektronen Synchrotron (DESY). As an endstation, we used a custom-made ultrahigh-vacuum (UHV) chamber which is equipped with iron and cobalt evaporators and a SPECS Phoibos 150 HV hemispherical analyzer with a delay line detector and wide-angle lens. This experimental setup allows us to perform hard X-ray photoelectron spectroscopy (HAXPES) measurements while growing thin CFO films. The CoFe_2_O_4_ thin films have been prepared on Nb-doped SrTiO_3_(001) single crystalline substrates using reactive molecular beam epitaxy (RMBE) in an UHV system at a base pressure of $10^{-8}\;m\mathrm{bar}$ in the deposition chamber. Since Nb-doped SrTiO_3_ is conductive this substrate is very appropriate for this experimental setup in order to reduce charging effects. The substrates used in this study (SurfaceNet GmbH) have a surface orientation tolerance less than $0.1^{\circ }$. Before deposition, the substrates were cleaned by annealing at $400\;{}^{\circ }C$ for 1 h in an oxygen atmosphere of $1\times 10^{-4}\;m\mathrm{bar}$. The RMBE deposition process is done in the following steps: before the start of the deposition, the substrate is heated by electron bombardement to a deposition temperature $T_{\mathrm{dep}}$ (400 °C in this work) and the UHV chamber is filled with molecular oxygen up to a partial pressure of $p_{O_{2}}$ (ranging from $1\times 10^{-7}$ to $5\times 10^{-6}\;m\mathrm{bar}$ in this work). In two molecular beam evaporators with closed shutter, pure cobalt and iron rods are heated to evaporation temperature by electron bombardment. In order to keep the growth rate constant, the ion flux leaving the evaporators is monitored using a fluxmeter. Once the flux has stabilized, co-deposition of the CoFe_2_O_4_ films is started by opening the shutters of both evaporators. After the intended evaporation time, the deposition is stopped by closing both evaporator shutters. Next, the sample heater is switched off, so that the substrate starts cooling down. At a substrate temperature of $100\;{}^{\circ }C$, the oxygen supply is cut and the UHV chamber returns to its base pressure. During growth, the Fe 2p and Co 2p core levels were rapidly measured (2 min per scan) by HAXPES measurements at an excitation energy of hν=4600 eV. After deposition, low-binding energy scans in the range from 0 eV to 140 eV and high resolution Fe 2p and Co 2p scans were performed in-situ.

Additionally, after film growth X-ray diffraction (XRD) experiments were performed ex-situ at beamline P08 of synchrotron radiation source PETRA III at DESY. For these measurements, a photon energy of 15 keV and a six-circle diffractometer with a two-dimensional PILATUS 100k detector was used.

**Figure 1:**
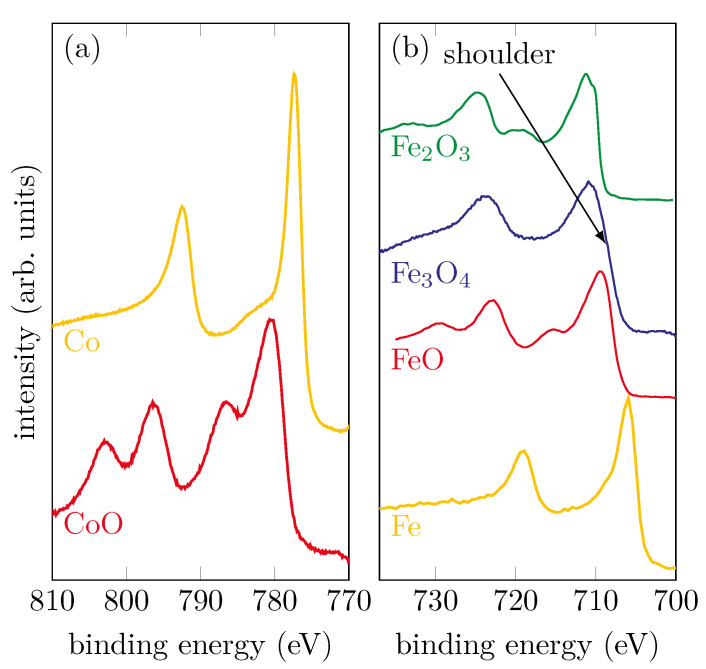
Reference spectra of (**a**) metallic Co and CoO samples and (**b**) metallic Fe and different iron oxide compounds.

Figure 1a shows reference spectra of metallic Co and CoO ultra thin films prepared by molecular beam epitaxy (MBE). In addition, Figure 1b shows the spectra of metallic Fe, as well as the various iron oxide compounds FeO, Fe_2_O_3_ and Fe_3_O_4_. The Fe spectrum stems from a 100 nm thin film prepared by MBE. The reference spectra of the iron oxides originate from single crystals cleaved in-situ directly before XPS measurements. Depending on the chemical composition, the spectra have characteristic shapes and properties. These are, as already described, the positions of the photoelectron peaks, as well as their satellites. Based on this, it is easy to make comparisons with the spectra obtained from prepared samples.

For metallic Co and Fe, it can be seen that there are no satellites and the peak shape is asymmetric. CoO, which consists only of Co^2+^ ions, has two shake-up satellites that are shifted to higher binding energies by 6.2 eV compared to the main peaks. For the iron oxides, the observation of the satellites, in addition to the different positions of the main peaks, provides information about the oxidation state. Considering FeO, which contains exclusively Fe^2+^ ions, a charge-transfer satellite can clearly be seen at still higher binding energies for both Fe 2p photoelectron peaks. If one compares this with Fe_2_O_3_, whose iron cations are exclusively trivalent, it can be seen that, in addition to different binding energies for the main peaks, the charge-transfer satellites are also shifted towards higher binding energies. For magnetite (Fe_3_O_4_), iron is present in both the Fe^3+^ and Fe^2+^ states. For this reason, the two satellites observed in the Fe_2_O_3_ and FeO spectra overlap and form a flat plateau between the main peaks of the Fe_3_O_4_ spectrum without any apparent satellite.

## 3. Results and Discussion

### 3.1. HAXPES

For this study, we prepared a series of three Co_*x*_Fe_3−*x*_O_4_/SrTiO_3_(001) samples grown under different conditions. Their oxygen partial pressures and growth rates are summarized in Table 1. In the following, we will separately examine them to investigate the influence of the growth parameters on the electronic structure. The spectra of all samples were calibrated to the binding energy of the O 1s peak $\left(E_{B}=530\;e\;V\right)$.

#### 3.1.1. Sample A—Low Oxygen Pressure

**Figure 2:**
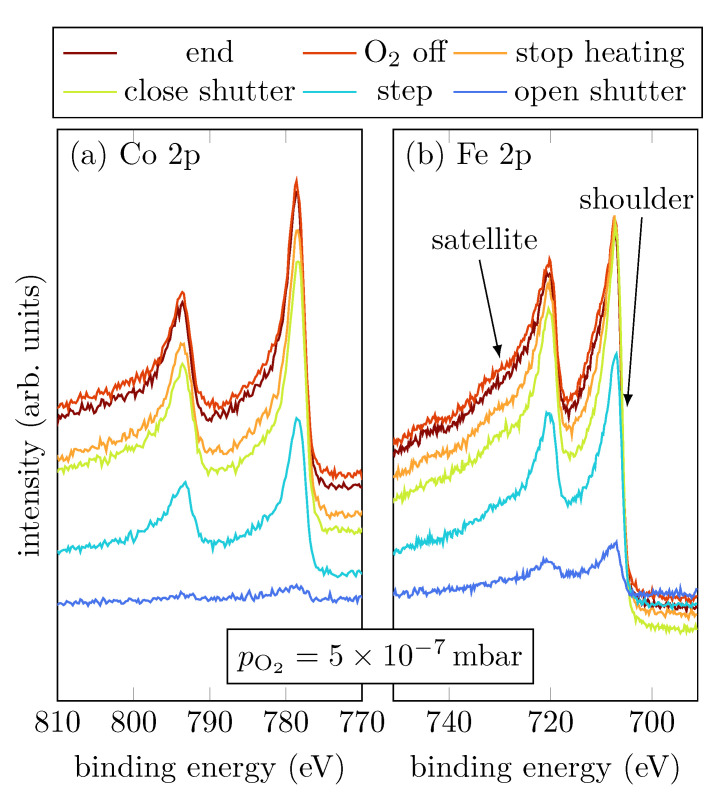
HAXPES spectra of sample A for characteristic events, as displayed, of the Fe 2p and Co 2p core-levels.

Sample A was grown with a low oxygen partial pressure of $5\times 10^{-7}\;m\mathrm{bar}$. Figure 2a,b show the Co 2p and Fe 2p peaks, respectively, at selected steps during the RMBE process.

The spectral shapes of both, Fe 2p and Co 2p, do not significantly change throughout the entire deposition process. In comparison with Figure 1, the last scan of the operando measurement shows that the spectra observed here indicate a major part of metallic Co and Fe as shown by the asymmetric peak shape. Nevertheless, oxidic contributions in Fe are recognizable, as indicated by the more pronounced shoulder and the emerging charge-transfer satellites at slightly higher binding energies than the main peaks.

**Figure 3:**
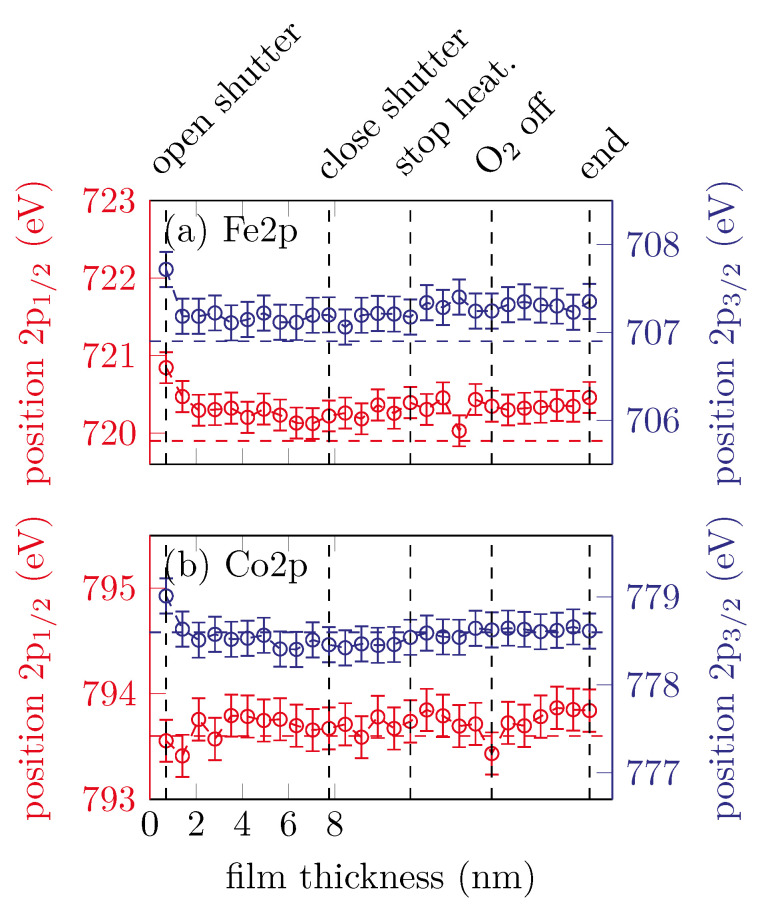
Peak positions of the (**a**) Fe 2p and (**b**) Co 2p peaks for sample A. The horizontal dashed lines represent the respective literature values for metallic Fe and Co.

Figure 3 summarizes the peak positions of the Fe 2p and Co 2p core-levels of all recorded spectra after fitting a Lorentzian function. It is obvious that all peaks except the Co 2p1/2 peak are shifted to lower binding energies within the very first scans. After the first few scans the peak positions remain constant within uncertainties. The Co 2p1/2 peak position remains constant from the very first scan. The observed peak positions of (793.6±0.4) eV for the Co 2p1/2 and (778.6±0.4) eV for the Co 2p3/2 peak agree well with the literature for metallic Co as reported by Betteridge [13]. For the Fe spectrum, the peak positions also agree well with observations for metallic Fe from the literature [14].

#### 3.1.2. Sample B—Intermediate Oxygen Pressure

**Figure 4:**
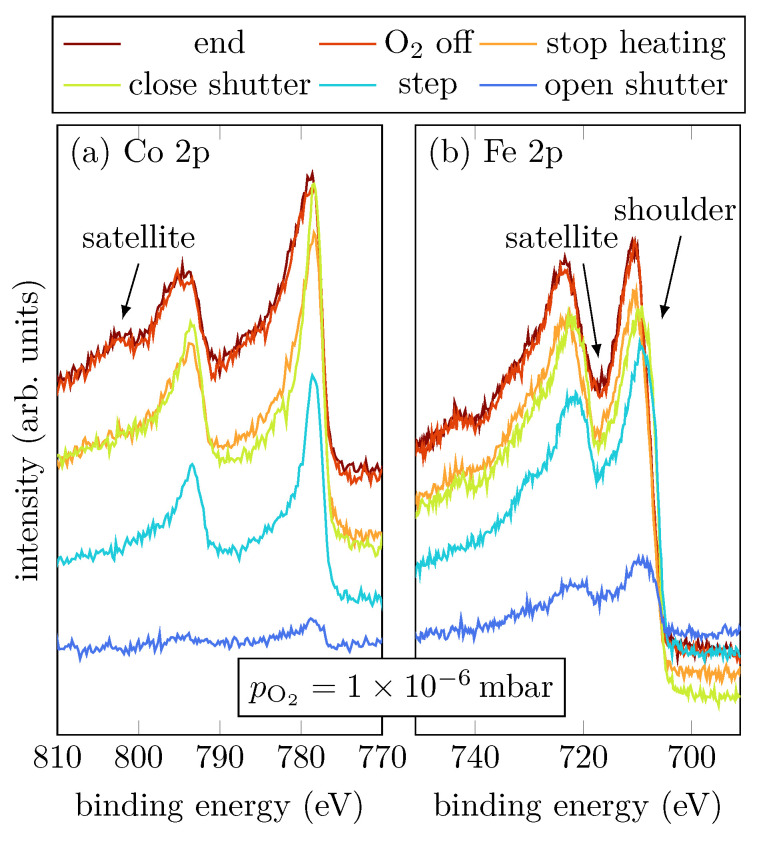
HAXPES spectra of sample B for characteristic events, as displayed, of the Fe 2p and Co 2p core-leveles.

Sample B was grown with an intermediate oxygen partial pressure of $1\times 10^{-6}\;m\mathrm{bar}$. Figure 4a,b show the Co 2p and Fe 2p peaks, respectively, at selected steps during the RMBE process.

During evaporation, the spectra show that the cobalt atoms are mostly in a metallic Co^0+^ charge state, indicated by the absence of charge-transfer satellites (cf. Figure 1a). On the other hand, Fe grows oxidic with an amount of Fe^2+^, indicated by the shoulder of the Fe 2p3/2 peak located at 705.8 eV (cf. Figure 4b) [15]. Once the shutter is closed, the Fe 2p3/2 and Fe 2p1/2 peaks are shifted to higher binding energies, which correlates to an increasing amount of Fe^3+^. Moreover, a charge-transfer satellite between the Fe 2p1/2 and Fe 2p3/2 peak at around 717 eV becomes visible after the shutter is closed, suggesting a light increase of the Fe^3+^ amount due to stronger Fe oxidation than for low oxygen pressure. After heating is turned off, it is clearly visible that also Co oxidizes, indicated by the emergence of the satellites of the Co 2p1/2 and Co 2p3/2 peaks. Furthermore, there is only one satellite for the Co 2p1/2 peak clearly visible at (801.4±0.8) eV, and the satellite for the Co 2p3/2 peak can only be guessed at around 786 eV (cf. Figure 4a).

**Figure 5:**
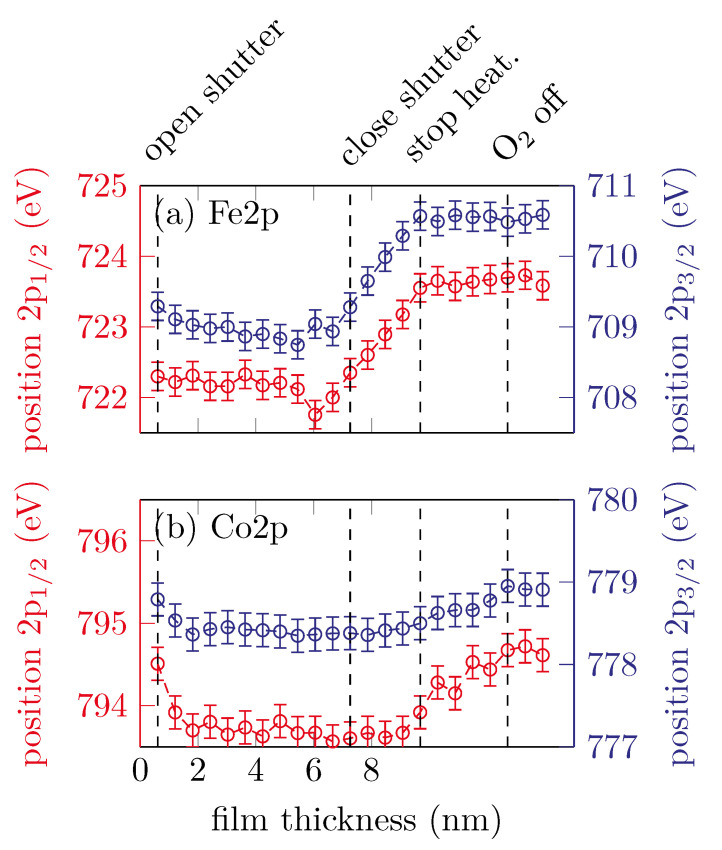
Peak positions of the (**a**) Fe 2p and (**b**) Co 2p peaks for sample B.

Figure 5 shows the peak positions of the Fe 2p3/2, Fe 2p1/2, Co 2p3/2 and Co 2p1/2 peaks changing with film thickness.

It is clearly visible that both of the Fe 2p peak position remain constant for approximately the first 7.26 nm until the shutter is closed and then shift to higher binding energies with increasing scans, which corresponds to an increasing amount of Fe^3+^ valence states. After the heater is turned off, the peak positions remain constant at around (710.8±0.4) eV for the Fe 2p3/2 and (723.7±0.4) eV for the Fe 2p1/2 peak. The peak positions for both the Co 2p3/2 and Co 2p1/2 peaks remain constant within uncertainties until the heater is turned off, at around 778.5 eV and 793.7 eV, respectively. This indicates Co^2+^ valence states. After the heater is turned off, the peak positions shifting to higher binding energies until the oxygen valve is closed.

#### 3.1.3. Sample C—High Oxygen Pressure

**Figure 6:**
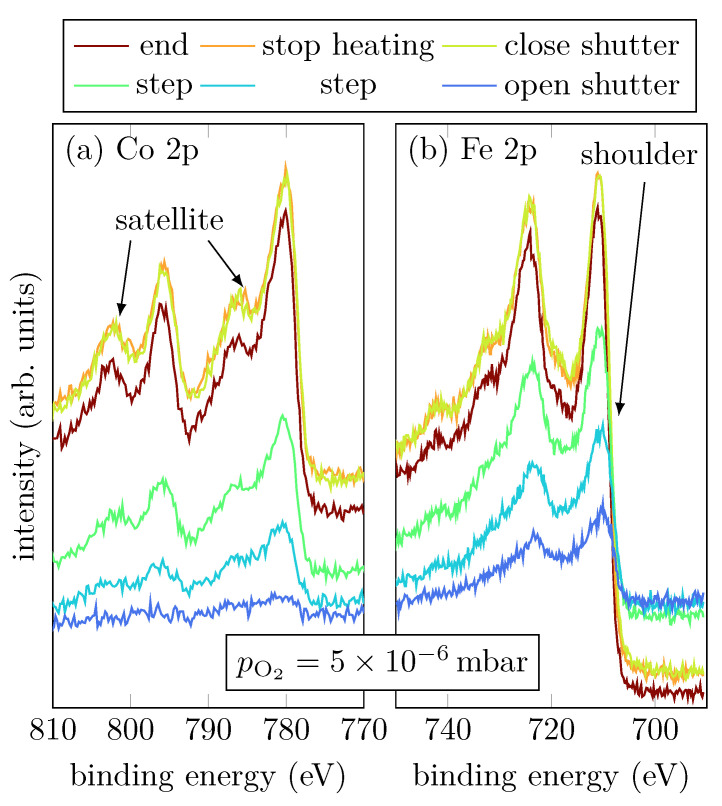
HAXPES spectra of sample C for characteristic events, as displayed, of the Fe 2p and Co 2p core-leveles.

Sample C was grown with a high oxygen partial pressure of $5\times 10^{-6}\;m\mathrm{bar}$. Figure 6a,b show the Co 2p and Fe 2p peaks, respectively, at selected steps during the RMBE process.

Looking at Co during growth, it is noticeable that immediately after the shutter is opened, the typical Co shape for Co^2+^ has formed. Two shake-up satellites of the Co 2p1/2 and Co 2p3/2 can be recognized, which are shifted to higher binding energies. The distance between main peaks and satellites is about (6.2±0.5) eV, which indicates a Co^2+^ valence state [16,17,18]. During the whole evaporation process the satellites become more and more pronounced.

Interestingly, during the growth only divalent Fe seems to be present. This is indicated by the shoulder of the Fe 2p3/2 peak. Initially, the spectral region between the Fe 2p3/2 peak and the Fe 2p1/2 peak does not exhibit any satellite structure, which indicates mixed valences of Fe^2+^ and Fe^3+^. However, starting at a film thickness of 4.6 nm, a satellite at 718.2 eV appears and becomes more and more pronounced with increasing thickness, but only in the spectra recorded after closing the shutter it is really pronounced indicating that Fe is only present in Fe^3+^ states.

**Figure 7:**
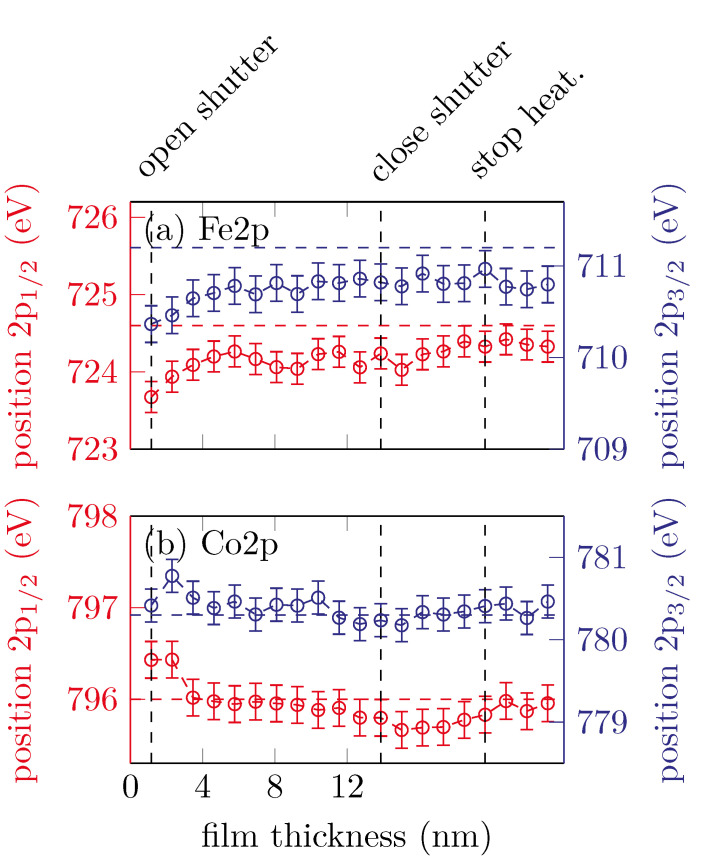
Peak positions of the (**a**) Fe 2p and (**b**) Co 2p peaks for sample C. The horizontal dashed lines represent the respective literature values for CFO.

Figure 7 shows the peak positions of the Fe 2p3/2, Fe 2p1/2, Co 2p3/2 and Co 2p1/2 peaks with respect to the film thickness.

The Fe peaks shifting to higher binding energies in the very first scans and then remain constant at around (710.8±0.5) eV and (724.3±0.5) eV for the Fe 2p3/2 and Fe 2p1/2, respectively. This is expected for the formation of cobalt ferrite with major Fe^3+^ content [16].

The Co 2p peak positions shift to lower binding energies with increasing film thickness. After the shutter is closed (12.6 nm) the peak positions remain constant at (780.3±0.4) eV and (795.8±0.4) eV for the Co 2p3/2 and Co 2p1/2, respectively. As mentioned before, this is an indicator for Co^2+^, as expected for cobalt ferrite.

For a quantitative analysis of the HAXPES data, the intensities $I_{\mathrm{Fe}}$ and $I_{\mathrm{Co}}$ of the Fe 2p and Co 2p have been numerically integrated. For this purpose, the spectra were subtracted by a Shirley background and the relative photoelectron yield $Y_{\mathrm{Fe}}$ has been calculated by using the formula

(1) $$Y_{\mathrm{Fe}}=\frac{I_{\mathrm{Fe}}/\sigma_{\mathrm{Fe}}}{I_{\mathrm{Fe}}/\sigma_{\mathrm{Fe}}+I_{\mathrm{Co}}/\sigma_{\mathrm{Co}}}.$$

The photoionization cross sections σ were taken from Trzhaskovskaya et al. [9] with respect to the used excitation energy $(\sigma_{\mathrm{Fe}}=4024b$ and $\sigma_{\mathrm{Co}}=4513b)$. For this sample we find a relative photoelectron yield of $Y_{\mathrm{Fe}}=0.644$. Using the relation $x=3Y_{\mathrm{Fe}}$, this leads to a stoichiometry of Co_1.07_ Fe1.93 O_4_. For samples A and B we find a relative photoelectron yield of 0.606 and 0.607, respectively.

### 3.2. XRD

**Figure 8:**
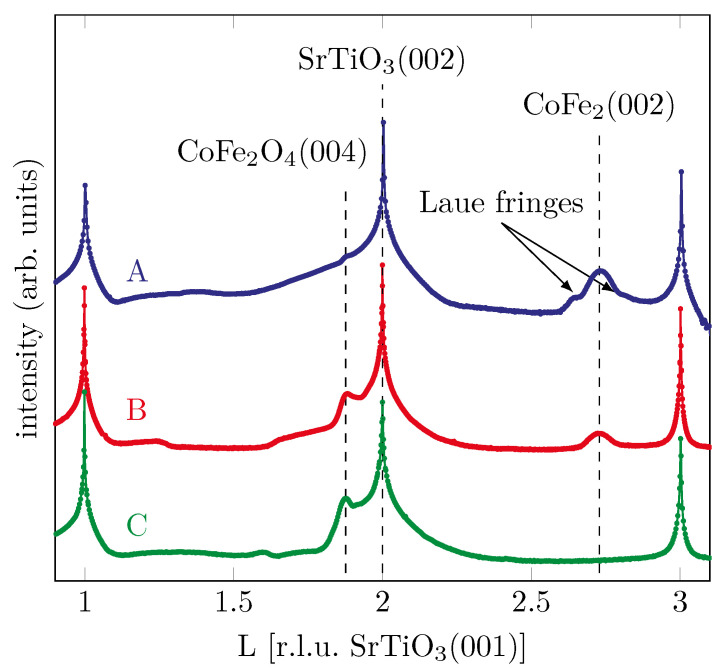
XRD measurements after film deposition of the different samples with varying oxygen partial pressure.

In order to control the structural properties of the samples, ex situ XRD measurements have been performed in θ−2θ geometry after growth of the films. All data were scaled to the reciprocal lattice units (r.l.u.) of SrTiO_3_(001). Figure 8 shows the recorded XRD scans for all prepared samples.

For sample A grown with the lowest oxygen partial pressure, we find an addtional diffraction peak at a *L*-value of 2.729, which is in good agreement with a Co-Fe-alloy (CoFe_2_(002)) as reported by Nishizawa and Ishida [19]. In addition, the first oscillation of very small Laue fringes, correlated to the peak of the Co-Fe-alloy, can be seen. This indicates a well-ordered crystalline film growth.

Comparing this to the sample with the intermediate oxygen partial pressure, we see that the peak at L=2.729 remains with lower intensity, again indicating a CoFe_2_ phase. In contrast to the sample discussed previously, a peak at L=1.881 can now also be found, which can be attributed to CFO while, in principle, it can also be due to Fe_3_O_4_ [20]. Thus, the film is characterized by coexistence of metallic and oxidic phases.

For the sample grown with the highest oxygen partial pressure, which already shows CFO behavior in the HAXPES measurements, a diffraction peak is also seen in the XRD at L=1.878, which points to the (004) Bragg reflex of CoFe_2_O_4_. The goal to produce crystalline epitaxial CFO is thus confirmed. For the samples with the medium and high oxygen partial pressure we find the (004) Bragg reflex of Fe_3_O_4_ and CFO at lower *L* values than the (002) Bragg reflex of the substrate due to its larger vertical atomic layer distances compared to SrTiO_3_(001).

## 4. Conlusions

In summary, three different Co_*x*_Fe_3−*x*_O_4_ samples with varying oxygen partial pressure were deposited on SrTiO_3_(001) substrates and chemically characterized in situ by means of time resolved operando HAXPES during film growth. For the lowest oxygen partial pressure of $5\times 10^{-7}\;m\mathrm{bar}$, the film grows almost exclusively metallic with only weak oxidic shoulders during the entire growth process. Increasing the oxygen partial pressure to $1\times 10^{-6}\;m\mathrm{bar}$, we see that cobalt first grows metallic and then oxidizes once heating is turned off. Iron grows first as Fe^2+^ states and then shows additional Fe^3+^ features after the shutter is closed. Increasing the oxygen partial pressure even further to $5\times 10^{-6}\;m\mathrm{bar}$ the film appears to grow strictly as cobalt ferrite from the very beginning, as aimed for for spintronic applications.

Additional XRD measurements confirm the results obtained by the HAXPES measurements very well. For the sample with the lowest oxygen partial pressure, a Bragg peak can be seen which indicates formation of crystalline Co-Fe alloy. For the sample with the medium oxygen partial pressure, the Co-Fe alloy peak is still visible and another peak appears indicating a cobalt ferrite content. For the sample with the highest oxygen partial pressure, metallic contributions disappear and CFO Bragg peaks appear exclusively, indicating the growth of a CFO film of high structural quality.

## Figures and Tables

**Table 1:** Overview of the prepared samples with the respective oxygen partial pressure and the growth rate.

Sample	$p_{\begin{array}{c} O_{2} \end{array}}$ (mbar)	Growth Rate (pm s${}^{-1}$)	Co Content *x*
A	$5\times 10^{-7}$	6.4	1.18±0.3
B	$1\times 10^{-6}$	5.5	1.18±0.3
C	$5\times 10^{-6}$	10.5	1.07±0.3

## Data Availability

The data presented in this study are available on reasonable request from the corresponding author.
